# Self-Processing and the Default Mode Network: Interactions with the Mirror Neuron System

**DOI:** 10.3389/fnhum.2013.00571

**Published:** 2013-09-11

**Authors:** Istvan Molnar-Szakacs, Lucina Q. Uddin

**Affiliations:** ^1^Semel Institute for Neuroscience and Human Behavior, University of California, Los Angeles, CA, USA; ^2^Tennenbaum Center for the Biology of Creativity, University of California, Los Angeles, CA, USA; ^3^Department of Psychiatry and Behavioral Sciences, Stanford University School of Medicine, Stanford, CA, USA; ^4^Department of Psychology, University of Miami, Coral Gables, FL, USA

**Keywords:** functional connectivity, embodiment, mentalizing, autobiographical memory, medial prefrontal cortex, posterior cingulate cortex

## Abstract

Recent evidence for the fractionation of the default mode network (DMN) into functionally distinguishable subdivisions with unique patterns of connectivity calls for a reconceptualization of the relationship between this network and self-referential processing. Advances in resting-state functional connectivity analyses are beginning to reveal increasingly complex patterns of organization within the key nodes of the DMN – medial prefrontal cortex and posterior cingulate cortex – as well as between these nodes and other brain systems. Here we review recent examinations of the relationships between the DMN and various aspects of self-relevant and social-cognitive processing in light of emerging evidence for heterogeneity within this network. Drawing from a rapidly evolving social-cognitive neuroscience literature, we propose that embodied simulation and mentalizing are processes which allow us to gain insight into another’s physical and mental state by providing privileged access to our own physical and mental states. Embodiment implies that the same neural systems are engaged for self- and other-understanding through a simulation mechanism, while mentalizing refers to the use of high-level conceptual information to make inferences about the mental states of self and others. These mechanisms work together to provide a coherent representation of the self and by extension, of others. Nodes of the DMN selectively interact with brain systems for embodiment and mentalizing, including the mirror neuron system, to produce appropriate mappings in the service of social-cognitive demands.

## Introduction

### Defining the self and brain networks for self-related processing

The importance of self-knowledge has been asserted by philosophers, religious leaders, and thinkers cross-culturally. The Chinese philosopher Lao-Tzu claimed: “*He who knows others is wise; He who knows himself is enlightened*.” The English cleric C. C. Colton wrote, *“He that knows himself knows others, […],”* emphasizing the importance of self-knowledge for the sake of understanding others, as did Gandhi, who wrote, “*He who knows himself, knows God and all others*” (Gandhi, 1955). Throughout history, several examples exist of thinkers who have realized that representations of the self and others are intimately intertwined – that the self is a social stimulus. Current psychological theories suggest that the self may be considered a “special” stimulus, but also imply that it has similarities to other familiar and non-familiar stimuli that can be considered on a continuum of “familiarity” (e.g., kin recognition; Platek and Kemp, 2009) and “knowledge” (e.g., self-knowledge; Klein et al., 2002). For example, simulation theory proposes that in order to understand others we look inside ourselves to mentally simulate how we might act in given social situations (Gordon, 1986). Conversely, Gallotti and Frith (2013) have recently suggested that in order to understand ourselves, we pay close attention to the social behavior of others.

One major and useful distinction that has guided research on the neural representation of the self is that between the physical and psychological aspects of the self (Gillihan and Farah, 2005). Physical aspects of the self are typically examined in studies of self-face recognition, body recognition, agency, and perspective taking. Psychological aspects of the self tend to be operationalized with studies examining autobiographical memory and self-knowledge or self-referential processing (SRP) of personality traits. This conceptual distinction bears out in neuroimaging work, which suggests that physical or embodied self-related processes and psychological or evaluative self-related processes rely on distinct yet interacting large-scale brain networks (Lieberman, 2007; Uddin et al., 2007; Molnar-Szakacs and Arzy, 2009; Molnar-Szakacs and Uddin, 2012). For the purposes of the current review, the principal neural networks we will consider are the default mode network (DMN) and the human mirror neuron system (MNS).

The repeated observation that the medial prefrontal cortex (MPFC), posterior cingulate cortex (PCC), lateral parietal cortices, and medial temporal lobes paradoxically exhibit high levels of activity during resting baseline and decreases in activity during externally oriented cognitive tasks led to the initial characterization of these regions as belonging to a “default mode” of human brain function (Shulman et al., 1997; Gusnard and Raichle, 2001; Raichle et al., 2001; McKiernan et al., 2003; Fransson, 2006). This set of regions is more active when individuals rest than when they are engaged in goal-directed tasks. Importantly, these cortical regions tend to fluctuate in a coherent manner – a phenomenon termed functional connectivity – which further supports the notion that they constitute a network of functionally related processing areas (Greicius et al., 2003; Fox et al., 2005; Golland et al., 2008). This network has also been referred to as the “task-negative network” (Fox et al., 2005), or the “cortical midline structures” (Northoff et al., 2006), and was originally proposed as a system for evaluating “information broadly arising in the external and internal milieu” (Raichle et al., 2001). The DMN has been posited to underlie a variety of general functions such as stimulus-independent (Mason et al., 2007) or task-unrelated thought (McKiernan et al., 2006), as well as social-cognitive or self-related processes, including episodic memory (Greicius and Menon, 2004), memory consolidation (Miall and Robertson, 2006), social processing (Iacoboni et al., 2004; Uddin et al., 2005), and various forms of self-related processing (Gusnard et al., 2001; Wicker et al., 2003b; Buckner and Carroll, 2007). More specifically, the DMN’s involvement is observed most consistently during the psychological task of reflecting on one’s own personality and characteristics (SRP), rather than during physical self-recognition (Qin and Northoff, 2011).

The MNS was first identified in non-human primates. Mirror neurons are active when an agent performs an action, and when it observes that same action being performed, in essence, creating an agent-independent connection between actor and observer (Rizzolatti and Sinigaglia, 2010). Based on the property of mirror neurons to internally simulate actions performed by others, it has been proposed that the MNS may provide the link between the physical representation of the self as related to the physical representation of others (Uddin et al., 2005, 2006, 2007). That is, when we see *another’s hand* grasping an object, we activate the regions of *our brain* that control grasping; when we hear sounds associated with *someone else’s action*, we activate the appropriate movement regions of *our brain*; and by extension, when we observe the *emotional states of others*, *we can feel the same emotion* in empathy (Carr et al., 2003; Gazzola et al., 2006; Molnar-Szakacs et al., 2006). These mirror-like processes are influenced by the observer’s perspective and the goal of the action itself, which appears to be even more important than the way in which an action is performed (Gazzola et al., 2007). The brain regions involved in creating these interpersonal links include the MNS and its associated regions – the inferior frontal gyrus (IFG)/premotor cortex (PMC), the anterior insula (AI), primary sensory and primary motor cortices, the inferior parietal lobule (IPL), and the superior temporal sulcus (STS).

The physical/psychological distinction, while perhaps simplistic, has facilitated the study of the neural networks underlying self-related processes. As the face is the most identifiable marker of the physical aspect of the self, it has been the subject of extensive study at the behavioral and neural levels. In particular, in our own work, we observed that the pattern of signal increases in the right IFG and right IPL were related to the amount of self-face presented in morphed stimuli (morphed with the face of a familiar other). In other words, the greater amount of “self” present in the stimulus, the greater the activation in right fronto-parietal regions (Uddin et al., 2005). These regions overlap the human MNS, whose role is to map the actions of others onto one’s own motor repertoire via a simulation mechanism (Rizzolatti et al., 1996). Similar findings have since been published (Sugiura et al., 2005; Platek et al., 2006; Uddin et al., 2006), supporting the role of the human MNS in physical self-recognition.

Psychological aspects of the self, such as those accessed through personality traits, likely evoke a representation of the self predominantly through linguistic aspects of the self-schema (Faust et al., 2004; Molnar-Szakacs et al., 2005b; Moran et al., 2006). Self-schemata are cognitive representations of the self that are derived from past social interactions and experiences and promote the elaboration of memories that may be used to guide future behavior (Markus, 1977). In one of the first neuroimaging studies on the subject, Kelley and colleagues used a trait adjective judgment task to compare processing of self-, other-, and case-referential adjectives. Results showed that the MPFC was selectively engaged in the self-related condition, while relevance judgments (i.e., “Does this adjective describe you/U.S. President George Bush?”), when compared to case judgments (i.e., “Is this adjective in lowercase letters?”), were accompanied by activation of the left IFG and the anterior cingulate cortex (ACC) (Kelley et al., 2002). This initial finding has since been replicated (Moran et al., 2009; Feyers et al., 2010), underscoring the role of MPFC in self-processing (Moran et al., 2013). Additionally, two recent meta-analyses have parcellated MPFC into ventral and dorsal aspects (Denny et al., 2012; Wagner et al., 2012), showing that ventral MPFC (VMPFC) responds more to self, and dorsal MPFC (DMPFC) responds more to others. Earlier work showed a similar dissociation along the lines of mentalizing about similar others (engaging VMPFC) and metalizing about dissimilar others (engaging DMPFC) (Mitchell et al., 2006).

Self-reference and self-relevance – whether by visual self-face recognition or through the enhanced memory for trait adjectives – invoke autobiographical memory processes (Molnar-Szakacs and Arzy, 2009). Memory is vital to the survival of the self, as we use our memory for past events to predict the future and update action plans in a flexible, goal-oriented manner (for reviews, see Schacter et al., 2007, 2008). Recently, neuroimaging studies have started to investigate the neural networks subserving self-projection in time (Addis et al., 2007; Buckner and Carroll, 2007; Szpunar et al., 2007; Arzy et al., 2008). Arzy and colleagues used a paradigm that involved participants making mental self-projections to both past and future events, and found an effect of self, whereby participants responded significantly faster to self-relevant (personal) events than to non-self-relevant (world) events. Self-location in time was shown to recruit a distributed neural network – including anterior temporal, occipito-temporal, and temporo-parietal regions – that partly overlaps the DMN (Arzy et al., 2008). These brain regions were also recruited in studies of visuo-spatial perspective taking and spatial self-location (Vogeley and Fink, 2003; Blanke et al., 2005; Arzy et al., 2006). In one of the first descriptions of the DMN, Raichle et al. (2001) proposed a domain-general role for the PCC in providing complex visual representations to consciousness.

Taking into consideration the many facets of self-relevant processing such as self-face recognition, personality trait judgments, and autobiographical memory, it is not surprising that these processes recruit a vast network of brain regions. These include the human MNS for physical aspects of self-relevant processing, as well as the MPFC node of the DMN during SRP and the PCC/precuneus node of the DMN for self-location in time and space. In order to bridge the gaps between these neural and psychological levels of analysis, we need to correlate cognitive and affective experiences of self with the underlying neural processes supporting them. Inspired by current and historical psychological theories (Gordon, 1986; Gallotti and Frith, 2013) and extending upon our previous work (Molnar-Szakacs et al., 2005b; Uddin et al., 2005, 2006; Molnar-Szakacs and Uddin, 2012), we propose that many of the same neural systems are engaged for self- and other-understanding. Thus, having privileged access to our own physical and mental states allows us to gain insight into others’ physical and mental states through the processes of embodiment and mentalizing. These cognitive processes are supported at the neural level by two large-scale, interacting networks – the MNS and the DMN, respectively. A more in-depth understanding of the functionally relevant nodes of each network, and the interactions between them, will help us advance toward a more complete theory of self-representation. By bringing together recent work on the fractionation of these complex networks, we aim to contribute to a more complete understanding of the self.

### Neural processes giving rise to the self

Preston and de Waal (2002) formalized a theory of emotional-motor resonance in the Perception–Action Model, which holds that perception of a behavior performed by another automatically activates one’s own representations for the behavior, and output from this shared representation automatically proceeds to motor areas of the brain where responses are prepared and executed. Emotional-motor resonance may also be called emotional empathy or embodied simulation – processes related to the same bottom-up, automatic, and evolutionarily early mechanism. Embodied simulation implies transforming perceived actions and emotions into our own inner representations of those actions and emotions. This process, supported by interactions between the MNS and the limbic system, is fast, automatic, and pre-cognitive, and is thought to support our ability to empathize emotionally (“I feel what you feel”) (Preston and de Waal, 2002). Current evolutionary evidence suggests that embodied simulation is a phylogenetically early system for empathy, and that there is also a more advanced cognitive perspective-taking (or theory of mind, ToM/mentalizing) system mediating empathic responses in humans (de Waal, 2008).

Higher-level cognitive empathy requires that we actively think about, or reflect on others’ actions and emotional states, including perspective taking or ToM/mentalizing (de Waal, 2008). Mentalizing refers to the process of understanding another person’s perspective, and appears to depend upon higher cognitive functions such as cognitive flexibility (Decety and Jackson, 2004). Singer (2006) has proposed that mentalizing allows us to *understand* mental states such as intentions, goals, and beliefs, while embodied simulation allows us to *share* the feelings of others. Low-level embodied processes and higher-level mentalizing processes integrate their signals such that stimuli are “mapped” onto internal representations and combined with information from memory to plan future behavior, select a response, and act. Neuroimaging studies have implicated distinct neural networks subserving embodiment and mentalizing processes (Shamay-Tsoory et al., 2004; Singer, 2006; Vollm et al., 2006; Hooker et al., 2008). Mentalizing processes appear to be centered on the MPFC node of the DMN, while embodied simulation processes are implemented by the MNS – limbic system network (Preston and de Waal, 2002; Gallese, 2005; Iacoboni and Dapretto, 2006; Iacoboni, 2009).

As previously discussed, the human MNS supports a simulation-based, motor resonance mechanism, whereby we understand the actions and emotions of others by “embodying” them ourselves. It has been suggested that mirror neurons are a kind of “neural wi-fi” that monitors what is happening in others. This system tracks others’ emotions, what movements they’re making, and what they intend, and activates in our brains precisely the same areas that are active in theirs. This puts us on the same wavelength and it does so “automatically, instantaneously and unconsciously” (Goleman, 2006). Neuroimaging studies have provided evidence in support of this notion, showing common neural signatures while experiencing disgust (Wicker et al., 2003a), touch (Keysers et al., 2004), or pain (Singer et al., 2004; Jackson et al., 2006) in oneself, and when perceiving the same feelings in others. Between-brain analyses have also provided evidence for neural resonance between individuals during social interactions (Schippers et al., 2010).

In thinking about the self and others, mentalizing representations (Barsalou, 1999, 2008) and embodied representations (Goldman and de Vignemont, 2009) serve as the foundations for making inferences about our own mind as well as others’ minds. Recent work has suggested that higher-level inference-based mentalizing processes are grounded in their interactions with lower-level embodied simulation-based processes (Barsalou, 1999, 2008; Goldman, 2006; Keysers and Gazzola, 2007; Goldman and de Vignemont, 2009). This predicts that brain regions involved in high-level inference-based mentalizing are integrating their signals with lower-level simulation-based systems (Keysers and Gazzola, 2007; Uddin et al., 2007), implying DMN–MNS interactions during self-relevant processing (Sandrone, 2013). In a recent study, Schippers and Keysers have shown using Granger causal analyses that rather than simply being a feed-forward system in which visual representations are transformed into motor programs through a temporal → parietal → premotor flow of information, the MNS acts as a dynamic feedback control system, and that during gestural communication there is information flow within the system from premotor to parietal and temporal cortices (Schippers and Keysers, 2011). Their findings lend strong support to the notion of dynamic interactions between the MNS and the DMN.

Here we expand on recent theories linking embodiment and mentalizing systems (Keysers and Gazzola, 2007; Uddin et al., 2007; Molnar-Szakacs and Arzy, 2009; Paulus et al., 2013; Sandrone, 2013), and propose that the MNS and the DMN are functionally connected and dynamically interact during social-cognitive processing. Simulation-based representations serve to scaffold conceptual representations that allow us to understand the self in its social context. By virtue of their differential patterns of connectivity, subdivisions of the DMN can interact with the appropriate brain systems, including the MNS, in the service of self-related and social-cognitive demands. In light of recent work fractionating the DMN (Uddin et al., 2009; Andrews-Hanna et al., 2010), we will discuss some examples of how these low- and high-level mechanisms critical for representing the self are subserved by dissociable subdivisions of this network. In addition, we will highlight brain regions that may serve as key hubs mediating interactions between the DMN and MNS.

## Different Aspects of Self-Related Processing

### Self-related processing in the physical domain

One of the most important ways to identify one’s own person is to recognize one’s face and distinguish it from other persons’ faces. Among the first to study the neural correlates of self-recognition in neurotypical adults, Keenan and colleagues provided behavioral (Keenan et al., 2000) and neural (Keenan et al., 2001) evidence for a right hemisphere bias in self-face processing. Subsequent functional Magnetic Resonance Imaging (fMRI) studies of self-face recognition described activations in lateral prefrontal cortex and parietal cortices during self-face recognition (Kircher et al., 2001; Platek et al., 2004, 2006; Sugiura et al., 2005). A recent review has highlighted the common finding of right frontal and parietal activations accompanying self-face viewing, especially when compared to other familiar faces (Devue and Bredart, 2011). Furthermore, a meta-analysis of studies of self-face recognition found that in addition to right fronto-parietal regions which overlap the human MNS, the right precuneus is a region that is also associated with this task (Platek et al., 2008). In our own work (Uddin et al., 2005), we provide clear evidence for a right hemisphere network including the IFG, IPL, superior parietal lobule, and inferior occipital gyrus activated by recognition of the self-face. The pattern of signal increases we observed in these areas as the stimuli contain more “self” suggest that these areas comprise a unique system extending beyond mere recognition of faces and play a particular role in self-face recognition. Perception of the self-face appears to involve a simulation-like mechanism that recruits right hemisphere MNS matching the face stimulus to an internal representation of the self. We proposed earlier that mirror areas may be more active for stimuli containing more “self” because their role is to establish communication between individuals via a simulation mechanism that maps actions of others onto one’s own motor repertoire, thereby making others “like me” (Meltzoff and Brooks, 2001). Thus, when one sees one’s own image, these mirror areas are more strongly activated because of the ease with which one can map oneself onto one’s own motor system (Uddin et al., 2005). Interestingly, we also observed similar brain activation patterns distinguishing the self-voice from other voices, suggesting that the right hemisphere MNS may contribute to multimodal abstract self-representation (Kaplan et al., 2008).

Our results also demonstrated decreased activity within the DMN (precuneus, MPFC, and posterior superior temporal gyrus) only during processing of “self” stimuli (Uddin et al., 2005). This pattern of results led us to propose that the “familiar other” stimuli triggered social representations, and thus the task-related deactivation was compensated during viewing of the “other” by an increase in activity due to social processing. Thus, the overall result is lack of deactivation for “other,” not a true activation. It is possible that during viewing of the “familiar other,” with whom the subjects have a positive social relationship, the subjects automatically activate social representations to a greater extent than when viewing the “self.” In summary, the generalized signal decrease in these DMN areas due to the task demands is offset in the “other” condition by triggering social-cognitive processing, which previously has been shown to engage these regions (Iacoboni et al., 2004). Thus, recognition of familiar others seems to also recruit midline structures that have previously been implicated in social processing (Saxe, 2006). Taken together, these results emphasize the importance of dynamic interactions between the MNS and the DMN during the processing of self-relevant information. The MNS appears to play an important role in physical self-recognition, while the DMN participates in situating the self in its social context relative to familiar others.

### Self-referential processing in the verbal domain

The self-reference effect (Symons and Johnson, 1997) is a unique encoding phenomenon, whereby memory for previously presented trait adjectives (e.g., happy) is better if they had been processed with reference to the self (e.g., “does happy describe you?”) than if they had been processed only for their general meaning (e.g., “does happy mean the same as optimistic?”). In other words, as traits are incorporated into the self-schema, subsequent memory for these trait words is increased (Rogers et al., 1977). Several studies have used the self-reference effect to investigate SRP in the verbal domain. Using statements delivered through the auditory domain, Johnson and colleagues compared judgments about one’s own abilities, traits, and attitudes (such as “I can be trusted”) to a semantic judgment task. The self-referential condition was associated with activation in the MPFC and the PCC relative to the control condition (Johnson et al., 2002). Using a slightly different paradigm, Kjaer and colleagues asked participants to mentally induce thoughts reflecting on one’s own personality traits and physical appearance. Once again, self-referential conditions induced activation in midline DMN regions including the MPFC and precuneus when compared to the non-self-referential conditions (Kjaer et al., 2002). They also observed increased functional connectivity between frontal and parietal midline regions during self-referential conditions. As evidenced by these studies, SRP in the verbal domain appears to recruit midline components of the DMN.

To tease apart the role of different subdivisions of the DMN in verbal SRP, Lou and colleagues used a combined PET-TMS approach. In the PET study, they used visually presented personality trait adjectives that were either related to the self, to the participants’ best friend, or to the Danish Queen (Lou et al., 2004). Retrieval of self-related adjectives induced activation in the DMPFC, the PCC/precuneus, the right and left IPL, the left ventrolateral prefrontal cortex, and the middle temporal cortex including the hippocampus. As in previous studies, analysis of functional connectivity revealed significant interaction between anterior (DMPFC) and posterior (PCC, precuneus) midline regions of the DMN. Transcranial magnetic stimulation over the medial parietal region caused a decrease in the efficiency of retrieval of previous judgments of the mental self as compared to retrieval of judgments of others, confirming that this region may be a nodal structure in self-representation, mediating interactions between the DMN and other lateral cortical regions (Lou et al., 2004).

### Self-referential processing in the memory domain

Self-referential processing in memory depends on the individual’s life history and involves the recollection of past experiences, as the retrieved episodic information is unique to an individual and is tied to a specific personal context (Ingvar, 1985; Craik et al., 1999). Episodic memory retrieval (EMR), on the other hand, also includes the retrieval of events that are characterized by low self-relevance. Behaviorally, the link between SRP and EMR is reflected in the so-called self-reference effect of memory, as discussed above (Rogers et al., 1977; Symons and Johnson, 1997). Further support for this link comes from neuroimaging investigations. EMR studies report activations in brain regions that are also identified by SRP tasks, including the MPFC, as well as the medial and lateral parietal cortex (Donaldson et al., 2001) (for reviews, see Cavanna and Trimble, 2006; Legrand and Ruby, 2009). Because these brain areas also show high neural activity during resting states, both SRP and EMR have been considered possible functions of the DMN (Buckner et al., 2008).

In a study designed to explore the similarity and dissociability of SRP and EMR, Sajonz and colleagues found that self-referential stimuli specifically activate the PCC/anterior precuneus, the MPFC, and an inferior division of the IPL. In contrast, EMR success specifically involves the posterior precuneus, the anterior prefrontal cortex, and a superior division of the IPL extending into the intraparietal sulcus and the superior parietal lobule. Overlapping activations can be found in intermediate zones in the precuneus and the IPL but not in the prefrontal cortex (Sajonz et al., 2010). These findings clearly demonstrate that distinct subdivisions of the DMN are recruited during SRP as compared with more general EMR. This is of particular interest in light of earlier studies associating the MPFC with autobiographical memory retrieval (Gilboa, 2004; Svoboda et al., 2006), retrieval of self-referential episodes (Zysset et al., 2002), retrieval of self-generated versus externally presented words (Vinogradov et al., 2008), and the self-reference effect of memory (Macrae et al., 2004). These processes have in common that they involve self-referential and memory components at the same time. The data of Sajonz and colleagues seem to suggest that the self-referential component particularly contributes to activations of the medial prefrontal node of the DMN observed in these studies.

A functional connectivity analysis performed on the data suggests a functional segregation within the PCC/precuneus for SRP and EMR, respectively. Activity in the SRP-related seed in the PCC/anterior precuneus correlated with the MPFC, dorsal ACC, fusiform gyrus, and superior parietal lobule during SRP. In contrast, activity in the EMR-related seed in the posterior precuneus was associated with the responsiveness in a distinct region in the dorsal anterior paracingulate cortex during EMR (Sajonz et al., 2010). Taken together, these findings shed light on the parcellation of nodes within the DMN, and suggest that there is a functional segregation within the precuneus during SRP and EMR. Activity in anterior precuneus appears to be associated with SRP, a more self-directed process, whereas activity in posterior precuneus is associated with EMR, a more social and outward-directed process. This anterior/posterior functional parcellation within the precuneus mirrors the dorsal/ventral subdivision of the MPFC, as discussed above.

## Neural Networks, Functional Connectivity, and the Self

### Findings from resting-state fMRI

The past several years have witnessed a resurgence in the use of fMRI to study not only regional activation patterns in response to specific stimuli, but also functional connectivity between-brain regions both during task performance and during resting states. This focus on brain connectivity has emerged as a natural consequence of recent advances in methods for acquiring and analyzing resting-state fMRI data, as well as efforts such as the Human Connectome project (http://www.humanconnectomeproject.org/). Functional connectivity measured from fMRI data is defined as “temporal correlations between remote neurophysiological events” (Friston, 1994), and is typically quantified by conducting correlation analyses between regional timeseries (Cole et al., 2010). Since the initial demonstration that coherent low-frequency fluctuations in blood-oxygen-level-dependent (BOLD) signal index functionally significant brain systems (Biswal et al., 1995), the use of resting-state fMRI to characterize brain functional organization has sky-rocketed. This approach has been used to understand how the DMN might be further divided into functional subsystems.

It is often difficult to ascertain the functional roles of brain regions from their selective activation during processing of specific stimuli or associated with specific cognitive demands. Resting-state connectivity approaches, unconfounded by ceiling and floor effects in task performance, can provide complementary information regarding the functional roles of brain regions. It has been known since the initial study by Greicius et al. (2003) that brain areas comprising the DMN (PCC, MPFC, lateral parietal cortices), show coherent low-frequency fluctuations. Several recent studies examining the resting-state or intrinsic functional connectivity of the DMN have provided evidence for considerable heterogeneity between distinct nodes of the network. For example, the PCC has been shown to have stronger negative correlations with anterior cingulate and insular cortices, whereas the MPFC shows stronger negative correlations with posterior parietal cortices (Uddin et al., 2009). Several previous studies have demonstrated default mode suppression during goal-oriented task performance, with failure to suppress default mode activity being linked to decreased activity in task-relevant regions and attentional lapses, or decrements in performance (Weissman et al., 2006). Heterogeneity of DMN nodes in terms of their functional connectivity suggests that different avenues may exist for communicating with other brain systems critical for self-related processing.

While the MPFC and PCC are considered core “hubs” of the DMN, some have suggested that the network can be fractionated into subcomponents. Recently, Salomon et al. (2013) have proposed that the inferior and posterior parietal aspects of the DMN can be further subdivided such that some show greater involvement in self-referential judgments than others. Andrews-Hannah and colleagues found that one subsystem including DMPFC, temporo-parietal junction (TPJ), lateral temporal cortex, and temporal pole, is more engaged when individuals make self-referential judgments about their present situation or mental states, whereas a different subsystem comprised of VMPFC, medial temporal lobes, IPL, and retrosplenial cortex is more active during episodic judgments about the personal future (Andrews-Hanna et al., 2010). Others have subdivided the PCC into ventral and dorsal subdivisions. Leech et al. (2011) found that as difficulty increases during an N-back task, ventral PCC shows reduced integration within the DMN, whereas dorsal PCC shows increased integration with the DMN as well as attention networks. Taken together, these studies suggest that the concept of the DMN as a homogenous network should be refined and updated to account for heterogeneous patterns of activation and connectivity observed within the regions comprising it. This reconceptualization of the DMN as consisting of multiple interacting subsystems has clear implications for theories of the network’s role in self-related cognition. In particular, the identification of possible “nodes of association” creating functional links enabling communication between the DMN and MNS are now beginning to be revealed. It has recently been demonstrated that certain brain regions constitute a “rich club” of organization in that they are highly connected hubs that are connected to other highly connected hubs (van den Heuvel and Sporns, 2011). We propose that such highly connected brain regions, including the PCC/precuneus and AI, may play a role in orchestrating dynamic interactions between the DMN and MNS.

### Functions and functional connectivity of DMN nodes

Although the precise functional properties of the DMN are not yet established, a growing number of studies implicate this network in various aspects of self-related processing. For example, the DMN is implicated during self-related evaluations (Northoff et al., 2006; Buckner and Carroll, 2007) voluntary actions (Goldberg et al., 2008), episodic memory (Spreng et al., 2009; Sestieri et al., 2011), and planning. Previous studies have revealed functional subdivisions within the DMN (Uddin et al., 2009; Andrews-Hanna et al., 2010; Sestieri et al., 2011) using either data driven parcellation methods (e.g., ICA, graph-analysis), or using specific tasks such as EMR. Within-region functional subdivisions in the DMN are also starting to be described as related to various neural processes including SRP and EMR (Andrews-Hanna et al., 2010; Sajonz et al., 2010; Kim, 2012) and cognitive control (Leech et al., 2011). In the following sections, we will describe some relevant studies that used a connectivity approach to explore DMN function and connectivity with the MNS and other brain regions during self-relevant processing.

Due to the overlap between brain regions involved in self-processing and regions that constitute the DMN (D’Argembeau et al., 2005; Schneider et al., 2008), some speak of a so-called “default self,” arguing that the self may be more or less identical with the resting-state activity observed in DMN regions (Gusnard et al., 2001; Wicker et al., 2003b; Beer, 2007). A recent meta-analysis of 87 self-related studies has lent further support to this idea (Qin and Northoff, 2011). In their meta-analysis, Qin and Northoff asked a two-part question – is neural activity in the DMN self-specific, and is self-specific activity related to resting-state activity? The specificity of the self (e.g., hearing one’s own name, seeing one’s own face) in the DMN was tested and compared across familiar (using stimuli from personally known people) and other (strangers and widely known figures) conditions. A large MPFC regions was recruited for the self condition when compared to the familiarity and other conditions. Concerning other midline regions, there was either regional overlap of activations between the self and familiarity conditions in the MPFC, or between the familiarity and other condition in the PCC (Qin and Northoff, 2011). This finding is in accordance with previous studies finding both self-specific and non-specific regions within the DMN during self-relevant processing (Gusnard et al., 2001; D’Argembeau et al., 2005; Schneider et al., 2008).

An interesting finding to emerge from the meta-analysis by Qin and Northoff (2011) was the recruitment of the right IFG, as well as the left AI during self-specific conditions. The role of the IFG as one of the anchors of the MNS and its role in self-relevant processing are well established (Molnar-Szakacs et al., 2005a; Uddin et al., 2005). As we have previously discussed, the right IFG seems to be responsive to self-face stimuli as well as one’s own voice (Uddin et al., 2005; Kaplan et al., 2008). The insula has also been associated with self-specific stimuli in recent studies (Enzi et al., 2009; Modinos et al., 2009), and forms an integral part of the neural network important for emotional empathy, embodiment, and simulation (Carr et al., 2003; Singer et al., 2004). As the insula is heavily involved in interoceptive stimulus processing (Craig, 2003), one may suggest that the co-activation between insula and the DMN may be crucial in constituting the self and assigning self-specificity to stimuli. It has recently been demonstrated that the right AI plays a causal role in switching between the DMN and executive control networks (Sridharan et al., 2008). It has been suggested that the AI serves to detect events that are salient to the individual and mobilize neural resources in the service of appropriate behavioral responses (Menon and Uddin, 2010). That self-related stimuli should invoke activation of the insula is not surprising in light of these findings. Pre-reflective representations of visceral states of the self, for instance, seem linked to activations in the posterior and/or middle insula. By contrast, midline structures become active when subjects are asked to introspect, reflect, and report these states (e.g., heartbeat) (Critchley and Harrison, 2013). The AI seems crucial in linking the more posterior insula with these midline structures. Thus, interactions between the DMN and the MNS through the functional connectivity of midline structures and the AI could mediate the ability to represent one’s bodily states to enable conscious reflection on those states (Keysers and Gazzola, 2007).

### Interactions between the DMN and MNS

The integration of function between the DMN and the MNS have been the focus of several recent proposals on the neural bases of self-related cognition (Keysers and Gazzola, 2007; Uddin et al., 2007; Molnar-Szakacs and Arzy, 2009; Molnar-Szakacs and Uddin, 2012; Paulus et al., 2013; Sandrone, 2013). The results of Qin and Northoff (2011) also lend support to the notion that the self emerges from the interaction of these two neural networks. Their meta-analysis showed recruitment of DMN regions, including the MPFC and PCC, as well as MNS regions, including the IFG and AI, both during self-relevant processing.

Lombardo et al. (2010) used a functional connectivity approach to investigate the nature of the interaction between high-level mentalizing systems and embodied simulation-based representations during mentalizing and physical judgments about the self and others. The areas of overlap of activation between self and other consisted of the MPFC, PCC, and bilateral TPJ as well as the left anterior temporal lobe along the middle temporal gyrus, left primary sensorimotor cortex, and cerebellum. With a factorial design, they were able to test the interaction effect of whether mentalizing or physical representations recruit distinct functional circuits for the self or other. Similar patterns of functional connectivity between self and other conditions suggested that mentalizing representations are distributed across similar neural systems with respect to self and other. Conjunction analyses revealed a self–other distinction within the neural circuitry for mentalizing whereby the MPFC was biased for SRP, and the PCC and the TPJ were biased for other-referential processing, as has previously been shown (Ruby and Decety, 2001; Saxe et al., 2006; Pfeifer et al., 2007). As opposed to the previous within-region functional subdivisions we have discussed for the dorsal/ventral MPFC or the anterior/posterior precuneus, self–other distinction in this study mapped onto fronto-parietal DMN regions. Taken together, the results of these studies show that in addition to broad cross-regional functional specializations, region-specific functional specializations exist within nodes of the DMN.

A particularly interesting result of the study was that several MNS regions, including IFG/PMC, primary somatosensory cortex, and the AI were sensitive to processing of both self and other. The role of somatosensory cortex in low-level shared representations of touch (Keysers et al., 2004; Blakemore et al., 2005), self-experienced pain (Singer et al., 2004), and action–perception mirroring (Gazzola et al., 2006; Nanetti et al., 2009) is well established. Thus, the observation that primary somatosensory cortex is also recruited for mentalizing about self and other suggests that low-level embodied simulative representations computed by this region are also important for the processes underlying higher-level inference-based mentalizing when compared with reflecting on physical characteristics (Lombardo et al., 2010). In fact, connectivity analyses revealed that these two systems were specifically linked during mentalizing more than during physical judgments, and this pattern of connectivity was apparent for both self and other conditions. Taken together, these results provide strong evidence of the integration of function between the DMN and the MNS. The authors conclude that “the tight link between high-level inference-based mentalizing systems and low-level embodied/simulation-based systems suggests that these two neural systems for social cognition are integrated in a task-specific manner for mentalizing about both self and other” (Lombardo et al., 2010).

**Figure 1:**
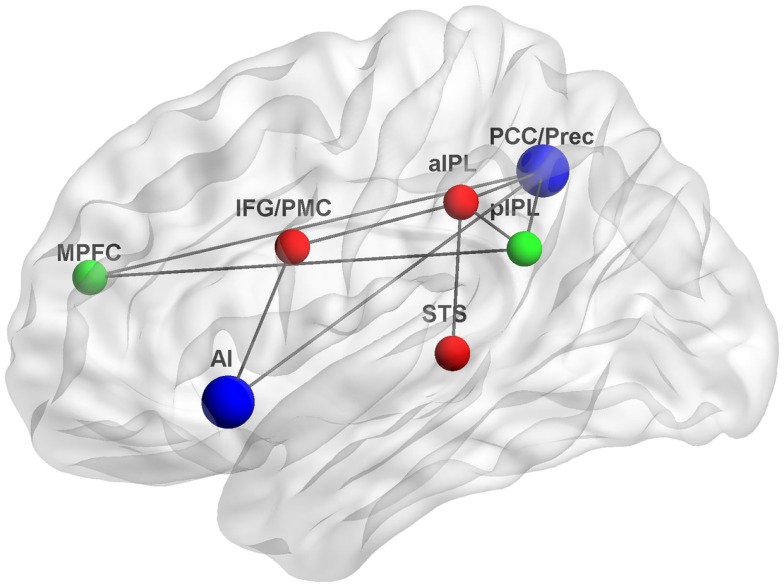
**Functional connections underlying interactions between the DMN and MNS**. The DMN, a system for psychological self-relevant processing and mentalizing, and the MNS, a system for physical self-recognition and embodied simulation, may interact through densely connected “hubs” such as the AI and PCC/Prec. Green, DMN nodes; red, MNS nodes; blue, interaction nodes; MPFC, medial prefrontal cortex, pIPL, posterior inferior parietal lobule; PCC/Prec, posterior cingulate cortex/precuneus; IFG/PMC, inferior frontal gyrus/premotor cortex; aIPL, anterior inferior parietal lobule; STS, superior temporal sulcus; AI, anterior insula; Gray lines indicate possible functional connections based on (Iacoboni et al., 2001; Lou et al., 2004; Iacoboni and Dapretto, 2006; Sridharan et al., 2008; Schippers and Keysers, 2011). Figure was created using BrainNet Viewer (http://www.nitrc.org/projects/bnv/).

The studies reviewed here suggest that interactions between the DMN and MNS during self-relevant processing may occur through several associated brain regions. Figure 1 depicts some of the possible neuroanatomical loci and functional connections underlying such interactions.

## Conclusion

Historically, scholars have pitted high-level inference-based mentalizing accounts and low-level embodied simulation-based accounts as opposites of each other (Gopnik and Wellman, 1992; Gordon, 1992). However, recent theories related to different aspects of self-representation have been focused on the possible integration of function between the DMN and the MNS (Keysers and Gazzola, 2007; Uddin et al., 2007; Molnar-Szakacs and Arzy, 2009; Molnar-Szakacs and Uddin, 2012; Paulus et al., 2013; Sandrone, 2013). Furthermore, interpretations of disturbances in self-relevant processing often invoke explanations that are based either in deficits of the DMN, the human MNS, or both. For example, theories of how we understand other minds have implicated both the DMN (Spreng and Grady, 2009) and the MNS (Gallese and Goldman, 1998); theories about moral cognition have been linked to both the DMN (Harrison et al., 2008) and the MNS (Molnar-Szakacs, 2011); and both the DMN and the MNS have been implicated in theories of physical self-representation (Uddin et al., 2007; Molnar-Szakacs and Arzy, 2009; Molnar-Szakacs and Uddin, 2012). In the realm of psychiatric or neurological disorders, both the DMN (Cherkassky et al., 2006; Uddin, 2011) and the MNS (Iacoboni and Dapretto, 2006; Molnar-Szakacs et al., 2009; Enticott et al., 2012) have been implicated in autism spectrum disorders and aberrant DMN connectivity and MNS dysfunction have been observed in schizophrenia (Garrity et al., 2007; Mehta et al., 2012). Taken together, this evidence from both the healthy and the atypical brain suggests that the human MNS and the DMN are functionally connected and are together profoundly implicated in social cognition that forms the basis of understanding the self. In the context of situations requiring understanding of others’ mental and physical states, such interactions facilitate the self–other mappings at the core of both embodiment and mentalizing processes.

Findings of functional specialization within the DMN are beginning to shed light on the ability of the network to support self-related processes as seemingly unrelated as autobiographical memory and verbal SRP. The findings reviewed here argue against viewing the DMN as a unitary system, and are compatible with the notion that the network consists of distinct, functionally specialized subsystems. It is becoming increasingly clear that great attention to anatomy can reveal subtle differences in circuitry of neighboring cortical regions of the DMN (Margulies et al., 2009). For example, we have seen that broad cross-regional functional specializations exist across regions of the DMN, such that the frontal MPFC node is more involved in self-related processing and the posterior PCC node is more involved in other-related processing. Additionally, region-specific functional specializations exist within nodes of the DMN, such that the VMPFC responds more to self and the DMPFC responds more to others. Furthermore, emerging findings from the functional connectivity literature can greatly inform theories of DMN involvement in self-related cognition. In particular, they highlight possible avenues for interactions between the DMN and MNS, and indicate how brain networks for mentalizing and embodiment might communicate. Indeed, the studies discussed above suggest that the DMN and MNS may interact at certain “rich-club” nodes, including the AI and the PCC. Through this interaction, embodied simulation-based representations serve to scaffold mentalizing-based representations. These representations allow the brain to construct a dynamic self, continuous through time, and able to plan for the future. A more in-depth understanding of the functionally relevant nodes of each network, and the interactions between them, will help us advance toward a more complete theory of self-representation in the brain.

## Conflict of Interest Statement

The authors declare that the research was conducted in the absence of any commercial or financial relationships that could be construed as a potential conflict of interest.
